# Association of Disease Knowledge and Medication Adherence Among Out-Patients With Type 2 Diabetes Mellitus in Khobar, Saudi Arabia

**DOI:** 10.3389/fphar.2020.00060

**Published:** 2020-02-20

**Authors:** Dhfer Mahdi AlShayban, Atta Abbas Naqvi, Othman Alhumaid, Ali Saad AlQahtani, Md. Ashraful Islam, Syed Azizullah Ghori, Abdul Haseeb, Majid Ali, Muhammad Shahid Iqbal, Mahmoud E. Elrggal, Azfar Athar Ishaqui, Mansour Adam Mahmoud, Irfanullah Khan, Shazia Jamshed

**Affiliations:** ^1^Department of Pharmacy Practice, College of Clinical Pharmacy, Imam Abdulrahman Bin Faisal University, Dammam, Saudi Arabia; ^2^College of Clinical Pharmacy, Imam Abdulrahman Bin Faisal University, Dammam, Saudi Arabia; ^3^Department of Clinical Pharmacy, College of Pharmacy, Umm Al Qura University, Makkah, Saudi Arabia; ^4^Department of Clinical Pharmacy, College of Pharmacy, Prince Sattam bin Abdulaziz University, Alkharj, Saudi Arabia; ^5^Pharmaceutical Research Center, Deanship of Scientific Research, Umm Al Qura University, Makkah, Saudi Arabia; ^6^Department of Pharmacy, King Abdulaziz Hospital, National Guard Health Authority, Alahsa, Saudi Arabia; ^7^Department of Clinical and Hospital Pharmacy, College of Pharmacy, Taibah University, Al-Madinah Al-Munawarah, Saudi Arabia; ^8^Department of Clinical Pharmacy, School of Pharmaceutical Sciences, University Sains Malaysia, Penang, Malaysia; ^9^Department of Pharmacy Practice, Kulliyah of Pharmacy, International Islamic University Malaysia, Kuantan, Malaysia; ^10^Qualitative Research-Methodological Application in Health Sciences Research Group, Kulliyyah of Pharmacy, International Islamic University Malaysia, Kuantan, Malaysia

**Keywords:** disease knowledge, medication adherence, patient compliance, concordance, diabetes mellitus, out-patients, Saudi Arabia

## Abstract

**Objective:**

The study aimed to evaluate the association between disease knowledge and medication adherence in patients with type 2 diabetes mellitus.

**Methods:**

A cross-sectional study was conducted for three months, in patients with type 2 diabetes who visited three community pharmacies located in Khobar, Saudi Arabia. Patients’ disease knowledge and their adherence to medications were documented using Arabic versions of the Michigan Diabetes Knowledge Test and the General Medication Adherence Scale respectively. Data were analyzed through SPSS version 23. Chi-square test was used to report association of demographics with adherence. Spearman’s rank correlation was employed to report the relationship among Hb_A1c_ values, disease knowledge and adherence. Logistic regression model was utilized to report the determinants of medication adherence and their corresponding adjusted odds ratio. Study was approved by concerned ethical committee (IRB-UGS-2019-05-001).

**Results:**

A total of 318 patients consented to participate in the study. Mean Hb_A1c_ value was 8.1%. A third of patients (N = 105, 33%) had high adherence and half of patients (N = 162, 50.9%) had disease knowledge between 51% - 75%. A significantly weak-to-moderate and positive correlation (ρ = 0.221, p < 0.01) between medication adherence and disease knowledge was reported. Patients with >50% correct answers in the diabetes knowledge test questionnaire were more likely to be adherent to their medications (AOR 4.46, p < 0.01).

**Conclusion:**

Disease knowledge in most patients was average and half of patients had high-to-good adherence. Patients with better knowledge were 4 to 5 times more likely to have high adherence. This highlights the importance of patient education and awareness regarding medication adherence in managing diabetes.

## Introduction

Diabetes mellitus (DM) is a type of chronic illness that requires careful management with medications to keep blood glucose level in recommended range (Abahussain and El-Zubier, 2005; World Health Organization, 2010; Abbas et al., 2015). The disease could result in micro and macro vascular complications that have serious short and long-term repercussions. Estimation of glycated hemoglobin (Hb_A1c_) is done to monitor the disease and forms the basis of treatment recommendations (Nazir et al., 2016). Patient adherence to anti diabetic medication therapy results in better control of disease and may help to keep Hb_A1c_ in recommended range (Al Dawish et al., 2016). There is a plethora of studies that highlight the importance of adhering to medications in type 2 diabetes mellitus (Al-Nozha et al., 2004; Abahussain and El-Zubier, 2005; Abbas et al., 2015; Al Dawish et al., 2016). However, it is imperative to improve patients’ disease knowledge to achieve high adherence to therapy (Yeh et al., 2018).

Patient education with an emphasis on adherence to anti diabetic medications and how it contributes positively to disease, would empower them to become compliant to prescribed therapy and, recognize and self-manage disease symptoms at home. Conversely, low literacy regarding disease is associated with poor treatment outcomes and a higher cost of therapy (Al-Nozha et al., 2004). Therefore, assessment of disease knowledge might be helpful in uncovering one of the determinants of poor disease outcome. Moreover, its relationship with adherence also provides an estimate of the extent to which knowledge translates into patients’ efforts for achieving treatment goals (Fawad et al., 2014; Abbas et al., 2015). Medication adherence in diabetic patients may be defined as the extent to which a patient remains committed to taking anti diabetic medications in right dose and frequency (Al Dawish et al., 2016). Patients and healthcare providers have a role to play in improving medication adherence (Brown and Bussell, 2011). Studies have emphasized that improvement in disease knowledge go hand-in-hand with improved medication adherence (Zullig et al., 2015).

Figures from International Diabetes Federation (IDF) report that globally there were more the 400 million patients living with diabetes in 2015. The World Health Organization estimates that there will be over 592 million patients with type 2 DM in 2035 (World Health Organization, 2016). The prevalence of type 2 DM in Saudi Arabia was 18.5% and has increased during the past decade (International Diabetes Federation, 2019). It remains as one of the main causes of death and disability in the country (Hussain et al., 2014; Sami et al., 2017). Saudi Arabia has the second largest diabetic population in the Gulf region and seventh largest in the world (Al Dawish et al., 2016). Evidence indicates that DM is more prevalent in urban areas and in males (Al-Nozha et al., 2004). The crude death rate is estimated to be 2.25% (95% CI: 2.02–2.5%) and accounts for 4.78% of total years lived with disability (95% CI: 3.86–5.7%) (Institute for Health Metrics and Evaluation, 2017) Ministry of Health KSA (2014).

Previous researches have highlighted a low adherence to medication therapy, poor self-management and an unsatisfactory disease knowledge in Saudi patients with diabetes. However, none of the studies investigated the relationship between disease knowledge and medication adherence (Abahussain and El-Zubier, 2005; Al-Aboudi et al., 2016; Alanazi et al., 2017).

## Methods

### Objective

The study examined the association between disease knowledge and medication adherence in patients with type 2 diabetes. The study also analyzed relationship between Hb_A1c_ (%) as a proxy for disease control, with adherence and disease knowledge.

### Duration and Venue of Study

A cross-sectional study was conducted for three months in three community pharmacies located in Khobar city of Saudi Arabia. Community pharmacies from three districts of the city were randomly selected.

### Target Population and Eligibility Criteria

All adult male and female out-patients, with or without comorbidities, who had established diagnosis of type 2 diabetes mellitus at least three months before the study, were identified as target population for this study. Patients not fulfilling the above criteria and those with an acute illness, diabetes complication and/or planned surgery were considered ineligible.

### Participants’ Recruitment

There were three types of patients who visited pharmacies for their medication needs; patients who obtained medicines through government supply, patients with corporate insurance and, patients who paid out-of-pocket cost. All patients had a medical record number (MRN). This MRN was obtained from patients and entered into the pharmacy software to retrieve electronic prescriptions. In addition to being used to check patients’ eligibility, the electronic patient records provided Hb_1Ac_ related data. Following confirmation of diabetes, they were briefed about the study. Those who agreed to participate were handed a written informed consent form. Patients who signed the consent were included in the study.

### Sampling Procedure and Sample Size

Convenience sampling procedure was employed to obtain information from patients who visited the community pharmacies. Data collection was done at a time of convenience, i.e., on weekends during evening hours. This time was selected based on peak visiting hours. The sample size was calculated based on disease prevalence with help of an online sample size calculator (Sampsize, 2019). The prevalence of DM according to IDF Report was 18.5% (International Diabetes Federation, 2019). This figure was entered in the calculator keeping a two-tailed alpha error rate of 0.05%, confidence level was kept at 95% and precision was set at 5%. The required sample size was 232 patients. A drop-out of 30% was added and final sample size required was 302. Post hoc power was calculated and was reported >85% (Cohen, 1988; Hertzog, 2008; Zhang et al., 2019).

### Research Instrument

The research instruments used for evaluation of patient’ disease knowledge and medication adherence were the Arabic versions of the Revised Diabetes Knowledge Questionnaire (DKT) and, the General Medication Adherence Scale (GMAS) respectively (Collins et al., 2011; Alhaiti et al., 2016; Fitzgerald et al., 2016; Naqvi et al., 2018; Naqvi and Hassali, 2019; Naqvi et al., 2019a; Naqvi et al., 2019b). The Arabic version of the Revised Diabetes Knowledge Questionnaire (DKT-2) is a 23-item questionnaire containing multiple choice questions (MCQs) to assess the knowledge of diabetes mellitus. It is a validated tool to measure disease knowledge with good internal consistency. The scale assesses a patient’s knowledge regarding the disease, complication, diet, treatment and monitoring, etc. (Alhaiti et al., 2016). The General Medication Adherence Scale (GMAS) was recently developed and validated in Saudi patients with chronic diseases. It contains 11 items and each item have 4 options (Naqvi et al., 2019a). The scale measures adherence to medications considering patient’s behaviors, comorbidities and out-of-pocket expenditures. Each option awards a score and sum of all individual scores yields a patient’s adherence to medication (Naqvi et al., 2018; Naqvi et al., 2019a; Naqvi et al., 2019b).

### Data Analyses

The data obtained were analyzed through SPSS version 23 and expressed as sample counts (N) and percentages (%). The data were checked for distribution and outliers by informal methods (Dunn and Clark, 1987; Islam et al., 2016). Based on data distribution, descriptive statistics as mean (X) and standard deviation (SD) were used for normally distributed data while median (M) and interquartile range (IQR) were used to report non-normally distributed data. Percentiles were used to report categorical data. Chi-square (χ^2^) test and cross tabulation was used to examine the association between patient demographics and medication adherence. Spearman’s rank correlation (r) was employed to report the relationship between disease knowledge and medication adherence. Adherence was assessed using multivariable logistic regression models, adjusting for patient baseline characteristics by using significant variables obtained from the Chi-Square (χ^2^) tests. The reliabilities of the GMAS and DKT questionnaires were analyzed using Cronbach alpha (α) (Cronbach, 1951; Cohen, 1988).

### Ethical Approval and Consent

The study was approved from the Institutional Review Board of Imam Abdulrahman Bin Faisal University (IRB-UGS-2019-05-001). Permission was also obtained from the pharmacies. A written informed consent was obtained from patients before participation.

## Results

A total of 318 patients consented to participate in the study. The reliabilities of GMAS and DKT questionnaires were reported at 0.81 and 0.75 respectively, i.e., satisfactory. The mean age of patients was 44 ± 15.5 years. The majority was male (N = 216, 67.9%), married (N = 231, 72.7%) with an income above SAR 10,000 (N = 150, 47.2%). Slightly less than half (N = 147, 46.2%) were university graduates. More than a third of patients had 1 – 3 comorbidities (N = 147, 46.2%). More than a third of patients (N = 123, 38.7%) were prescribed at least two medicines. Most patients were on insulin therapy (N = 225, 70.8%). More than half of patients had government insurance (N = 189, 59.4%) (**Table 1**).

**Table 1:** Demographic information (N = 318).

Demographic information	Sample (N)	Percentage (%)
Gender		
Male	216	67.9
Female	102	32.1
Marital status		
Married	231	72.7
Single	87	27.3
Monthly family income		
Less than SAR 5000 (i.e., < USD 1332.7)	54	17
Between SAR 5000 to 7500 (i.e., USD 1332.7 to 1999.2)	36	11.3
Between SAR 7500 to 10000 (i.e., USD 1999.2 to 2665.5)	78	24.5
Above SAR 10000 (i.e., > USD 2665.5)	150	47.2
Education level		
Primary education	60	18.9
Secondary education	111	34.9
Graduation	147	46.2
Comorbidity		
No comorbidity	171	53.8
Yes	147	46.2
Medicines per prescription		
Single medicine	105	33
Two medicines	123	38.7
Up to three medicines	69	21.7
Four or more medicines	21	6.6
Prescribed insulin therapy		
Yes	225	70.8
No	93	29.2
Health insurance		
Government insurance	189	59.4
Company insurance	69	21.7
Self-payment (No insurance)	60	18.9

1 USD equals SAR 3.75.

Out of total 147 patients with comorbidities, 30.6% had one comorbidity (N = 45), 60.5% had 2 comorbidities (N = 89) and 8.9% had three comorbidities (N = 13). Those who had one comorbidity (N = 45, 30.6%) mainly had a disease of cardiovascular origin along with DM. Those who had two comorbidities (N = 89, 60.5%), had cardiovascular + other endocrine diseases for most part and, to some extent, cardiovascular + pulmonary diseases, cardiovascular + musculoskeletal diseases, and, cardiovascular + liver/kidney diseases. Those who had 3 comorbidities (N = 13, 8.9%), had a combination of either cardiovascular + other endocrine diseases + liver/kidney disease or, cardiovascular + other endocrine diseases + musculoskeletal diseases, etc.

The average adherence score was 25.3 out of total 33 [median 27, IQR 7]. A third of patients (N = 105, 33%) had high adherence followed by same number of patients who were partially adherent. The average score for DKT-2 was 56.46 ± 16.7 out of 100. Most patients (N = 162, 50.9%) had disease knowledge score between 51–75%. (**Table 2**).

**Table 2:** Patients scores.

Adherence and disease knowledge scores	Sample (N)	Percentage (%)
GMAS adherence score		
High adherence 30 – 33	105	33
Good adherence 27 – 29	81	25.5
Partial adherence 17 – 26	105	33
Low adherence 11 – 16	21	6.6
Poor adherence 0 – 10	6	1.9
Diabetes Knowledge Test (DKT) score percentiles		
Between 76–90% correct answers	27	8.5
Between 51–75% correct answers	162	50.9
Between 25–50% correct answers	117	36.8
Less than 25% correct answers	12	3.8

**Figure 1:**
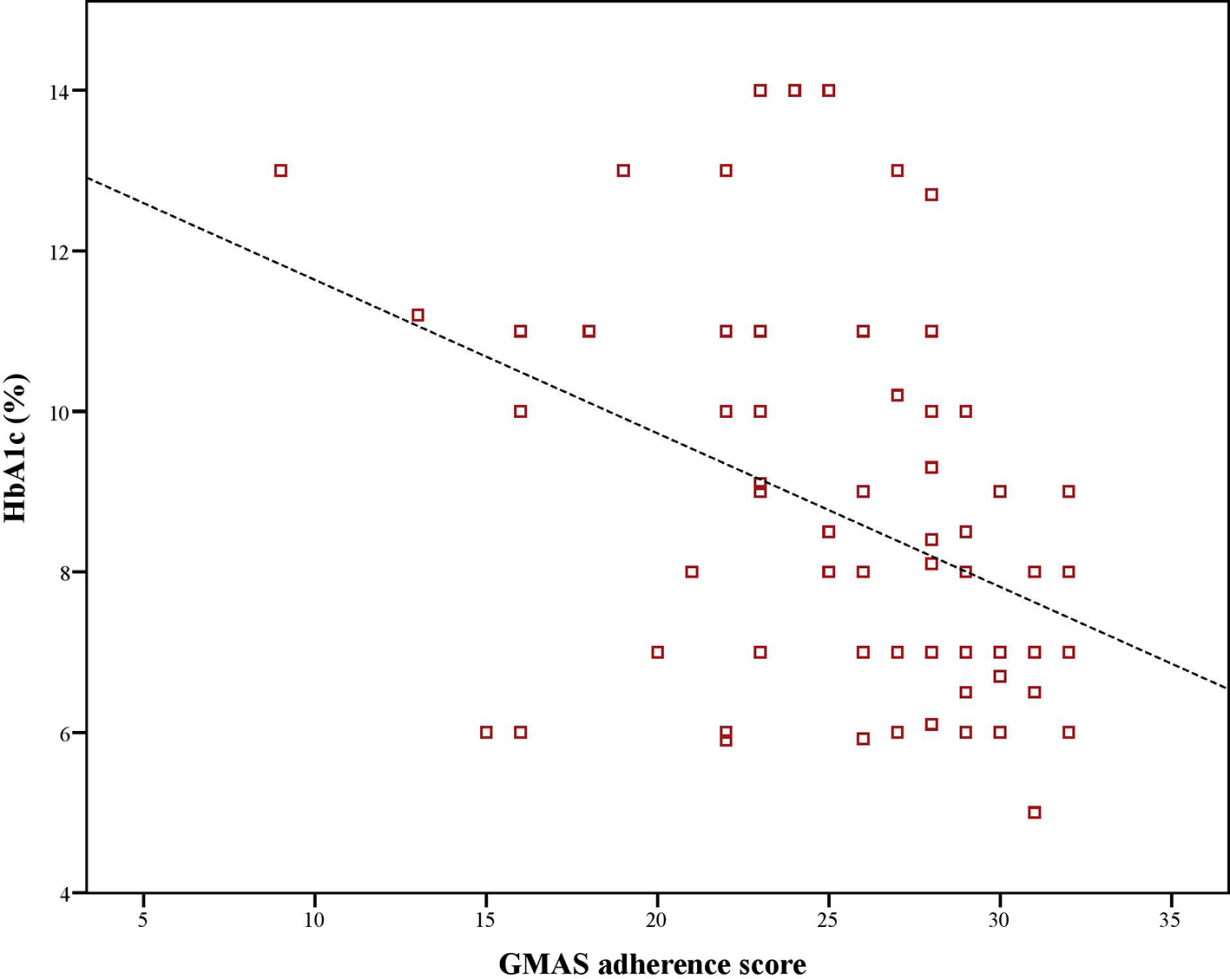
Correlation between adherence score and Hb_A1c_.

**Figure 2:**
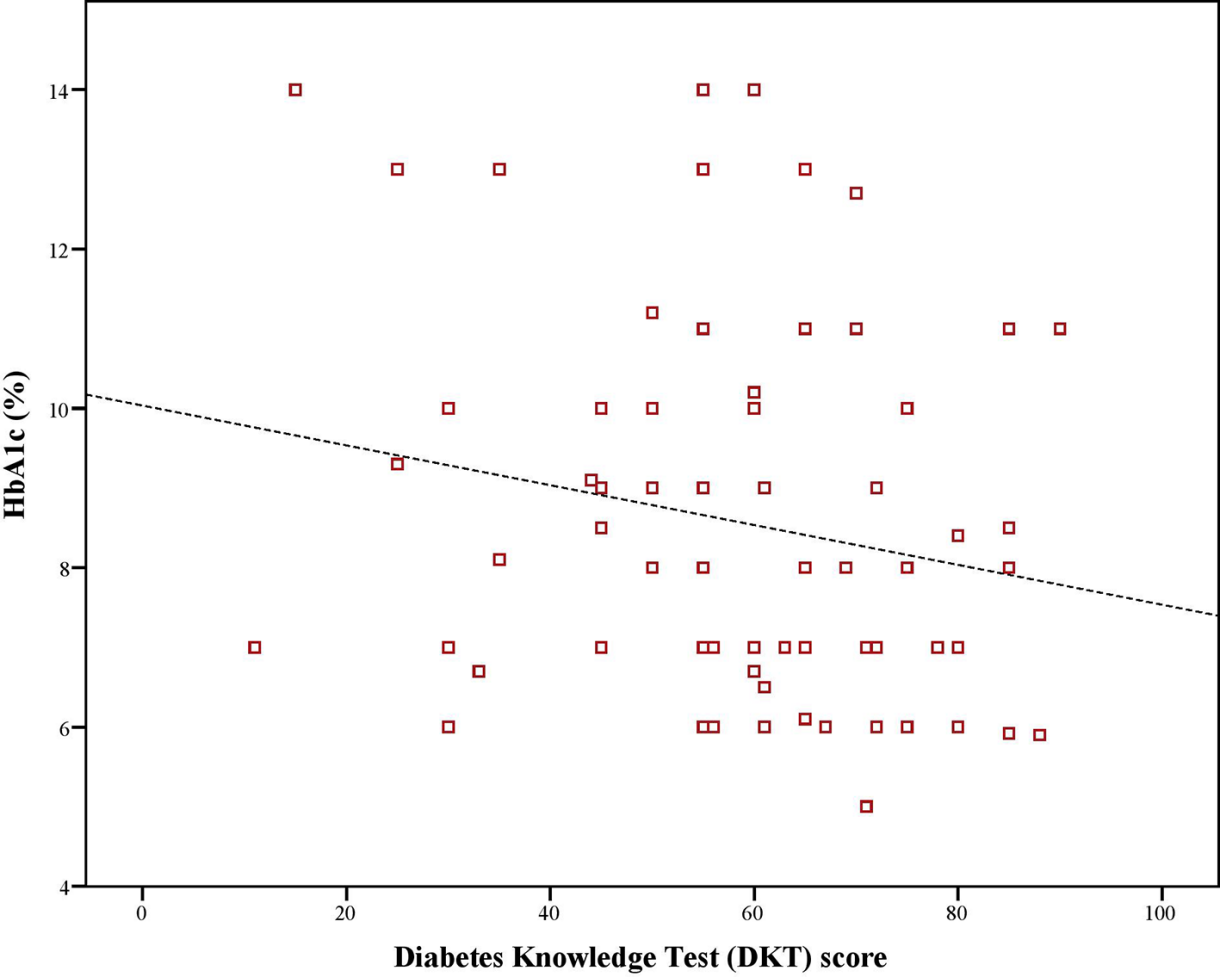
Correlation between Hb_A1c_ and disease knowledge score.

**Figure 3:**
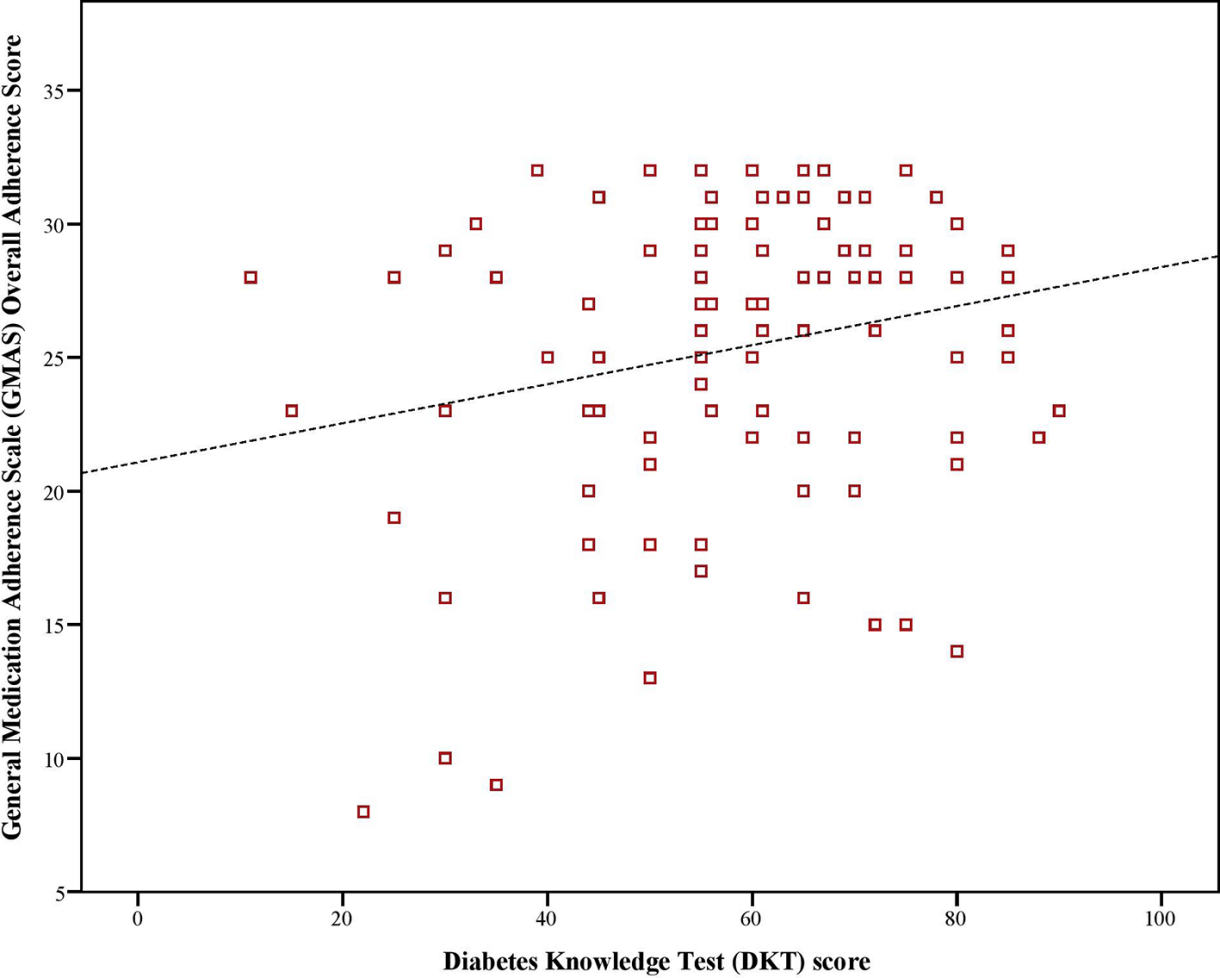
Correlation between adherence and disease knowledge scores.

Mean Hb_A1c_ value was 8.1%. There was a significant, negative, moderate-to-strong relationship between adherence score and, Hb_A1c_ values (ρ = –0.423, p < 0.01). Similarly, there was a significant, negative and weak-to-moderate relationship between Hb_A1c_ values and disease knowledge (ρ = –0.199, p < 0.01). Moreover, there was a significant, positive, weak-to-moderate correlation (ρ = 0.221, p < 0.01) between adherence and disease knowledge scores of diabetic patients (**Figures 1**–**3**).

There was a significant association between GMAS adherence percentiles and monthly family income (χ^2^ = 56.85, p < 0.01). The patients with a monthly family income less than SAR 5000 were observed in higher numbers in partial, low and poor adherence percentiles as compared to patients with higher income. Similarly, a significant association existed between adherence and education level (χ^2^ = 46.02, p < 0.01) as graduates were mostly observed to have a high adherence compared to patients who had primary or secondary education. Besides, a significant association was observed between adherence and mode of obtaining medicines (χ^2^ = 23.97, p < 0.01) as patients who obtained their medications by out-of-pocket expenditure were mostly seen in lower adherence percentiles.

There was a significant association between adherence and comorbidity (χ^2^ = 19.6, p < 0.01) as patients with comorbidity were mostly observed in high and good adherence percentiles. A significant association was reported between adherence and number of medicines per prescription (χ^2^ = 51.65, p < 0.01) as most patients prescribed with 2 medicines were mostly in high adherence percentile. Statistical significance was not achieved for association between adherence percentiles and; gender as well as, use of insulin (**Table 3**).

**Table 3:** Cross tabulation of dependent variables with GMAS score percentiles.

Monthly family income	GMAS adherence percentiles					P-value
	High adherence	Good adherence	Partial adherence	Low adherence	Poor adherence	
	Observed count (Expected count)					
Gender						>0.05
Male	72 (71.3)	54 (55)	72 (71.3)	12 (14.3)	6 (4.1)	
Female	33 (33.7)	27 (26)	33 (33.7)	9 (6.7)	0 (1.9)	
Monthly family income						<0.01*
< SAR 5000	6 (17.8)	12 (13.8)	24 (17.8)	9 (3.6)	3 (1)	
SAR 5000 - 7500	18 (11.9)	9 (9.2)	6 (11.9)	3 (2.4)	0 (7)	
SAR 7500 - 10000	15 (25.8)	27 (19.9)	27 (25.8)	9 (5.2)	0 (1.5)	
> SAR 10000	66(49.5)	33 (38.2)	48 (49.5)	0 (9.9)	3 (2.8)	
Education						<0.01*
Primary education	18 (19.8)	27 (15.3)	12(19.8)	3 (4.0)	0 (1.1)	
Secondary education	27 (36.7)	27 (28.3)	36 (36.7)	15 (7.3)	6 (2.1)	
Graduation	60 (48.5)	27 (37.4)	57 (48.5)	3 (9.7)	0 (2.8)	
How do you obtain diabetic medicines						<0.01*
Government supply	60 (62.4)	45 (48.1)	75 (62.4)	9 (12.5)	0 (3.6)	
Insurance	30 (22.8)	15 (17.6)	15 (22.8)	6 (4.6)	3 (1.3)	
Out-of-pocket cost	15 (19.8)	21 (15.3)	15 (19.8)	6 (4)	3 (1.1)	
Use of insulin						>0.05
Yes	69 (74.3)	54 (57.3)	84 (74.3)	15 (14.9)	3 (4.2)	
No	36 (30.7)	27 (23.7)	21 (30.7)	6 (6.1)	3 (1.1)	
Comorbidity						<0.01
Comorbidity present	51 (53.5)	54 (41.3)	42 (53.5)	9 (10.7)	6 (3.1)	
No comorbidity	54 (51.5)	27 (39.7)	63 (51.5)	12 (10.3)	0 (2.9)	
Medicines per prescription						<0.01*
1 medicine	21 (34.7)	24 (26.7)	48 (34.7)	9 (6.9)	3 (2.0)	
2 medicines	66 (40.6)	24 (31.3)	24 (40.6)	6 (8.1)	3 (2.3)	
3 medicines	12 (22.8)	27 (17.6)	24 (22.8)	6 (4.6)	0 (1.3)	
4 or more medicines	6 (6.9)	6 (5.3)	9 (6.9)	0 (1.4)	0 (0.4)	

SAR = Saudi Arabian Riyal, 1 USD equals 3.75 SAR, *Fisher Exact test.

Further analysis using multiple logistic regression revealed that patients who had a monthly family income above SAR 10,000 were five times more likely to be adherent (AOR = 5.4, p < 0.01) compared to patients with income lower than SAR 5,000. Besides, patients with comorbidity were two to three times more likely to be adherent to their medications (AOR = 2.7, p < 0.01). Moreover, patients who obtained their medications from insurance were four times more likely to be adherent as compared to those patients who paid out-of-pocket. On the negative side, patients with primary education were less likely to be adherent (AOR = 0.3, p < 0.01) compared to patients who were graduates. Finally, patients who answered more than 50% correct answers in the diabetes knowledge test questionnaire were observed to be 4–5 times more likely to be adherent to their medications (AOR 4.46, p < 0.01). The model was adjusted for age, income, education comorbidity and method of obtaining medicines, to amend for potential confounder of the relationship between disease knowledge and medication adherence. The model for medication adherence is tabulated in **Table 4**. In multiple logistic regression, “Enter” method was applied, multicollinearity was checked and was not found. Hosmer-Lemeshow test value was reported at χ2 = 11.334, p = 0.183 while Nagelkerke R Square value was 0.497.

**Table 4:** Model for medication adherence.

Variables	B	S.E.	P value	Adjusted OR	95% CI of OR	
					Lower	Upper
Age	0.031	0.012	0.007	1.032	1.009	1.056
Monthly income			0.000			
SAR Less than 5,000 (R)	–	–	–	–	–	–
Between SAR 5,000 to 10,000	1.420	0.465	0.000	4.155	3.695	13.185
SAR Above 10,000	1.686	0.473	0.000	5.400	4.718	18.763
Education level			0.000			
Graduation (R)	–	–	–	–	–	–
Primary level	-1.098	0.462	0.017	0.333	0.135	0.825
Secondary level	1.176	0.592	0.047	3.241	1.015	10.350
Comorbidity						
No (R)	–	–	–	–	–	–
Yes	1.002	0.360	0.005	2.724	1.346	5.515
Medicine obtain from			0.000			
Out-of-pocket (R)	–	–	–	–	–	–
Government supply	1.026	0.387	0.008	2.791	1.307	5.958
Company insurance	1.393	0.581	0.000	4.028	3.849	7.594
Diseases knowledge						
Less than 50% correct answers (R)	–	–	–	–	–	–
Between 50% to 100% correct answers	1.496	0.320	0.000	4.465	2.385	8.362

AOR, Adjusted odds ratio; CI, Confidence Interval.

## Discussion

Several studies have been conducted on measuring adherence to medications as well as knowledge regarding disease in Saudi patients with diabetes however, studies that examine the link between adherence and disease knowledge are lacking. This study was novel in this aspect and reported a weak-to-moderate positive relationship between the two. Moreover, it further revealed a moderate-to-strong negative relationship between adherence score and glycated haemoglobin A1c value as well between disease knowledge and same. Hb_A1c_ was considered as a proxy for disease control as the American Diabetes Association (ADA) mentions a better Hb_A1c_ value as an indicator for adequate glycemic control over 4 months (Sherwani et al., 2016). This approach has been previously used by AlQarni et al. (2019) in Saudi patients with T2DM. All correlations were statistically significant. This implied that patients who had better adherence and disease knowledge demonstrated better glycemic control. Moreover, it further highlighted that knowledge about the disease and adherence to therapy were related. Better knowledge contributes to better adherence. This finding was in line with previously reported literature that mentions disease literacy as a determinant of achieving positive treatment outcomes (Yeh et al., 2018; AlQarni et al., 2019). Our findings are in line with the results of Nazir et al. (2016) as there was a negative relationship between Hb_A1c_ value and, adherence and disease knowledge in patients with diabetes in Pakistan. Moreover, we also found disease knowledge as a determinant of adherence as patients with more than average knowledge of diabetes were 4 to 5 times more likely to be adherent to medications. In another study, patients with Hb_A1c_ values less than 6.5% had better disease knowledge and adherence (Al-Qazaz et al., 2011). In a systematic review, Kardas et al. (2013) reported that disease knowledge was a determinant of persistence. Better disease knowledge results in improved symptom recognition and self-management. It empowers patients to understand the importance of adherence and consequences of non-adherence (Zullig et al., 2015). Based on health behavior theory, patients would choose a behavioral option that helps them achieve a healthy status which in this case would be adhering to prescribed therapy that improves glycemic control (Becker, 1974; Nazir et al., 2016).

The scores for disease knowledge and adherence reported for Saudi patients were not satisfactory as less than 60% had high-to-good adherence and majority had average disease knowledge. This increases the likelihood of negative disease outcomes such as micro and macro vascular complications namely cardiovascular diseases, eyes and kidney damage, etc. Besides, it could also result in cerebrovascular events such as stroke. All these outcomes contribute to disability, morbidity, increase economic burden and lost productivity. These outcomes could worsen the health-related quality of life of diabetic patients and may increase likelihood of mortality in severe cases. Moreover, psychological impact of such outcomes may result in the form of depression. In this context, a study in Pakistan reported undiagnosed depression in diabetic patients (Abbas et al., 2015). Hence, this knowledge barrier could be a reason that diabetes remains as one of the main causes of death and disability in Saudi Arabia (Institute for Health Metrics and Evaluation, 2017).

Increasing health literacy remains a challenge in Saudi population. Recently, a large sample size study highlighted that more than half of Saudi population had low health literacy. The study stressed on the need to design and execute health literacy programs and campaigns (Abdel-Latif and Saad, 2019). Quite the reverse, Mashi et al. (2019) reported that there was high health literacy that was not associated with glycemic control, in Saudi patients with type 2 diabetes. Our study results contradict the findings of Mashi et al. (2019). This was evident in this study as most patients were graduates but had unsatisfactory disease awareness. An average Hb_A1c_ value of 8.1% explains that despite being educated, the patients’ diabetes were not adequately controlled. Moreover, most patients were on insulin therapy which further strengthen this proposition. This finding was in line with the work of Rabba et al. (2017). The study further highlighted that individual patient characteristics such as education, income, insurance and comorbidities may act as determinants of medication adherence. This occurrence was in line with previous study by AlQarni et al. (2019). However, the fact that education does not act as a determinant of disease awareness in this population uncovers the need to initiate diabetes awareness campaigns to educate patients about the disease, its symptoms and self-management that includes adherence to treatment. All healthcare professionals need to play a role in patient education.

Pharmacists fill prescriptions for patients after consultations. They are last healthcare professional patients see before leaving the hospitals. Pharmacist provide routine drug information service in Saudi hospitals (Alamri et al., 2017). Therefore, educational interventions by pharmacist could be more beneficial as compared to interventions by other HCPs. Moreover, pharmacists provide pharmaceutical care in which one of the core areas is patient education (Shah et al., 2016; Naqvi et al., 2019c). Studies that evaluate the benefits of an educational intervention by pharmacists to improve disease knowledge and adherence are recommended. The use of convenience sampling might have made it difficult to generalize the findings however, the demographics obtained in the current study were quite similar to the results of study by AlQarni et al. (2019) that used random sampling. Nonetheless, the results should be interpreted with caution.

## Conclusion

The disease knowledge in most patients was average and half of patients had high-to-good adherence. A significant weak-to-moderate correlation between disease knowledge and medication adherence was present. Moreover, there was a significantly moderate-to-strong, negative relationship between Hb_A1c_ and, disease knowledge as well as adherence. This revealed that glycemic control was better in patients with good knowledge of diabetes and high adherence to anti diabetic medications. A positive relationship between disease knowledge and adherence score was observed that highlights the impact of disease awareness on treatment concordance. This may result in better control of disease. These results highlight the importance of patient education and awareness regarding medication adherence in managing diabetes.

## Author’s Note

This manuscript is based on undergraduate research thesis submitted by AA and OA; students of Pharm.D 5^th^ Year for partial fulfillment of the degree of Doctor of Pharmacy (Pharm.D) at College of Clinical Pharmacy, Imam Abdulrahman Bin Faisal University, Dammam 31441, Saudi Arabia. No funding was sought for the study. The work was a collaborative assignment of Evidence Based Improvement (EBI) initiative team and supervisors from Saudi and Malaysian universities (Abbas, 2014; Naqvi, 2016).

## Data Availability Statement

All datasets generated for this study are included in the article/supplementary material.

## Ethics Statement

The studies involving human participants were reviewed and approved by the Institutional Review Board, IAU (IRB-UGS-2019-05-001). The patients/participants provided their written informed consent to participate in this study.

## Author Contributions

DA, AN, OA, and AA conceived the idea and designed the study. AA and OA formulated initial draft of introduction and DA and AN finalized it. SG, AH, MA, MSI, ME, and AI conducted literature review and added their part in the introduction. The methodology was jointly written by all authors as they were involved in data collection. Data was entered by DA, AN, OA, AA, and SG. Data analyses was carried out by AN, SG, MM, IK, and SJ. The re-analysis of data in the revised version was done by MAI. The results section were written by OA, AA, MM, and IK and were re-checked by SJ. The results section in revised version was modified by AN and MAI. The discussion and conclusion was written by MM, IK, AN, MA, AH, MSI, ME, and AI. During revision of manuscript all authors provided their insights. The whole process was supervised by SJ.

## Conflict of Interest

The authors declare that the research was conducted in the absence of any commercial or financial relationships that could be construed as a potential conflict of interest.
